# Annotations

**Published:** 1900-04-07

**Authors:** 


### Annotations.

### Drawing and Medicine.

Few things would be likely to excite greater consternation, in the mind of an average medical student, than an authoritative addition to the number of things which he is required to learn. But every man has some leisure, and many men feel occasional difficulty as to the best method of employing it. To such we would recommend the careful consideration of a suggestion put forth by Dr. Tuttle, of Cambridge (Mass.) as to the great value of the art of drawing as an adjunct to medical and surgical study and practice. Dr. Tuttle's paper, which appears in the *Philadelphia Medical Times and Register*, is, perhaps, a little discursive; but he rightly calls attention to the effect of drawing as a stimulus to the art of correct and careful observation, and very happily quotes an observation of Agassiz, that "a pencil is one of the best of eyes." In other words, the student who attempts to make a drawing of anything will be compelled to notice many peculiarities which would be likely to escape a cursory glance. Dr. Tuttle dwells also on the usefulness to the practitioner of his own sketches of the salient points of cases which have been under his treatment, and which will often serve as records to which he may appeal in relation to others bearing some similarity to them. He further points out how much may be done by a diagrammatic sketch to render a diseased condition intelligible to a patient, and thus to smooth the way for co-operation in the treatment that is required. To all these grounds for the recommendation of drawing, we would add that it may be made an admirable means of training the hand, especially the left hand, into habits of prompt obedience to the will; and that there is no shorter road to surgical ambidexterity than the practice of drawing two equal and symmetrical curves at once, one on either side of a vertical line. And the pursuit, if carried to even a moderate degree of excellence, is a source of continual pleasure. It becomes a source of pictorial memoranda, which, if less exact, are usually infinitely more gratifying than any of those yielded by what has been mischievously termed the "foe to graphic" art.

### Classification in Workhouses.

A pathetic interest attaches to a statement made at the Central Poor Law Conference held last week, where one of the speakers said that of the privileges granted to some of the well-behaved aged poor in workhouses, one of the most highly prized is the possession of a locker. We have heard much about old age pensions, and many have asked, Why trouble about such things when modern workhouses are made so comfortable? But if we wish to recognise what workhouse life really means, we must think of what it would be at the end of a long life to have nothing of one's own—no little treasures, no little mementos—and, if one did possess such things, to have no place in which to keep them. The bald fact that the possession of a locker should be a privilege so highly prized by respectable and aged inmates to whom the workhouse is not a mere temporary lodging, but is the "home" in which they must spend the rest of their days, is to our minds a horrible commentary upon the cast-iron rules to which all alike must conform when once they enter upon that final stage of a poor man's life. Mrs. Fuller, reading a valuable and practical paper upon the condition of the aged poor in rural workhouses, pointed out how much might be done to promote the comfort of that class of inmates who were known as the aged deserving poor by a relaxation of certain rules, and by the adoption of a degree of classification which could easily be arranged without going to any large expense. She thought that if large wards were sub-divided into more home-like rooms, or into recesses, by fixed or movable screens, and if other little things of this sort were done, the elderly inmates would soon form into little circles of their own; and she pointed out, what is sadly true, how largely the lot of the aged poor in the rural workhouses rests in the hands of the officials. Thereby hangs the moral of the tale. Classification cannot be a matter of codes and written rules. It must depend upon the common sense and judgment of the officials, and Mrs. Fuller very properly urged that if boards of guardians were careful to appoint a sufficient number of humane officials, if they would pay them well and would encourage them to make small relaxations of the rules of the house in favour of the aged deserving inmates, much might be done to soften the last days of those who, often through no fault of their own, had fallen into such grievous poverty.

### The Open-air Treatment at Hampstead.

There are several points of considerable interest in the medical report appended to the annual report of the North London Hospital for Consumption, more especially in regard to the results obtained by the so-called open-air treatment. It appears that on each corridor two small wards are reserved for such cases as are not deemed suitable for this mode of treatment, but that except in these wards all windows and ventilators are kept wide open day and night, with the result that the temperature is very seldom more than a few degrees higher than the air outside. There are also beds on the balconies outside two of the wards, the patients being kept warm with extra blankets and hot bottles. Unless thought unsuitable all cases are treated on the "open-air principle," and in regard to what we have often pointed out as to the desirability of all sorts and conditions of men taking more pains than they do to breathe pure air at night, it is worth noting that a marked improvement took place in patients who, although not affected with tuberculosis, were yet submitted to the "open-air" treatment. Turning now to results, we see that of 183 cases under this treatment, 80 were "Much Improved" on discharge, many of them "showing no sign of lung trouble at all on leaving the hospital." The average gain in weight was $9\frac{3}{4}$ lb., or $1\frac{1}{4}$ lb. per week. Then come 59 cases "Distinctly Improved." Of these "the large majority, in addition to increase of weight and diminution of temperature, evinced marked decrease of active mischief in the lungs." The average increase of weight was $4\frac{1}{2}$ lb., or $\frac{3}{4}$ lb. per week. Thus 76 per cent. of the open-air cases fell into these two categories of "Much Improved" and "Distinctly Improved," which is very satisfactory. It is to be noted that the "new treatment" is not a matter of "open-air" alone. The butcher's bill has been increased, which goes for something.

